# Addressing weight stigma and communicating with patients

**DOI:** 10.20945/2359-4292-2025-0335

**Published:** 2025-11-24

**Authors:** Stuart W. Flint, Paula V. Sozza, Adrian Brown

**Affiliations:** 1 School of Psychology, University of Leeds, Leeds, UK; 2 Scaled Insights, Nexus, University of Leeds, Leeds, UK; 3 Departamento de Psicologia, Universidade de São Paulo, Ribeirão Preto, SP, Brasil; 4 Department of Clinical and Health Psychology, Autonomous University of Barcelona, Bellaterra, Spain; 5 Centre for Obesity Research, University College London, London, UK; 6 Bariatric Centre for Weight Management and Metabolic Surgery, University College London Hospital NHS Trust, London, UK; 7 UCLH Biomedical Research Centre, National Institute of Health Research, London, UK

**Keywords:** Weight stigma, weight bias, healthcare, health policy, health communication

## Abstract

Substantial evidence highlights the pervasive nature of weight stigma, reported by people of all ages and backgrounds. Weight stigma is experienced across the life course and in many settings across society. These harmful experiences may include verbal and physical behaviours, with long-lasting effects on mental and physical health. They may also impact the patient-practitioner when weight stigma is experienced in a healthcare setting, as well as reducing health seeking behaviour and avoidance of healthcare. It is therefore essential that weight stigma in healthcare is addressed given the important implications of these experiences in this setting, Interventions need to be longer term and given the widespread nature of weight stigma, change is needed throughout society from policy to practice. Thus, a whole system approach to weight stigma is needed to address the entrenched and often robust nature of weight stigma attitudes.

## INTRODUCTION

Evidence highlighting the prevalence, impact and implications of weight stigma has increased substantially. Despite the continued misconception that weight stigma can be beneficial, there is an abundance of evidence that demonstrates these experiences have a detrimental impact on people living with obesity both on mental and physical health outcomes as well as behavioural responses. For instance, research has shown that weight stigma experiences are associated with higher depression and anxiety, reduced self-esteem, increased cardiometabolic risk factors, weight gain, increased risk of self-harming and eating disordered behaviours (^1^,^2^). Studies have also indicated that people living with obesity respond maladaptively including avoidance of settings where stigma occurs such as schools and education centres, social and community events and healthcare (^3^). Using a narrative review approach, our aim was to examine the empirical evidence examining the impact of weight stigma in healthcare and its impact on patients living with obesity. In doing so, we offer a range of strategies that can be implemented to reduce weight stigma.

## WEIGHT STIGMA IN HEALTHCARE AND HEALTH POLICY

Weight stigma is prevalent among healthcare professionals and within healthcare settings (^4^-^6^). Additionally, multinational studies indicate a high prevalence of weight stigma reported by doctors (^5^), with this stigma associated with biased beliefs about obesity (^4^). An important factor to highlight is that obesity is widely diagnosed based on Body Mass Index (BMI). This reliance can lead to inappropriate guidance and stigma in treatment, despite BMI having been originally designed for population-level analysis rather than individual assessment and not specifically to assess health. Furthermore, BMI is a limited measurement that does not adequately represent different racial and ethnic groups. Nevertheless, BMI remains widely used in clinical practice among healthcare professionals, even serving as an eligibility criterion for procedures (^7^) and referral for weight management interventions. The limitations of BMI as an individual assessment and diagnostic criterion for obesity are well recognized today. Recently, a new definition and diagnostic criterion were published to distinguish between preclinical obesity-characterized by excess adiposity without illness-and clinical obesity, defined by excessive adiposity along with dysfunctions in organs and cells, as well as reduced quality of life (^8^).

The evidence clearly demonstrates the complexity of obesity and weight gain. However, a widely held belief persists that weight is entirely under individual control and that modifying it simply requires effort in making dietary changes, maintaining a caloric deficit, and exercising more. This erroneous belief reinforces weight stigma and negative stereotypes about obesity, portraying individuals as lazy, uncommitted, undisciplined, and lacking the willpower to engage in changes that would lead to weight loss (^1^).

Weight stigma in healthcare settings is present across various fields, including medicine, surgery, nursing, psychology, nutrition, and dietetics, among other specialties, even those focused specifically on obesity treatment (^1^,^9^-^11^). Many patients report an excessive focus on weight and the promotion of weight loss strategies during consultations, with professionals disregarding their actual health concerns (^10^). Proper education on obesity is limited from the early stages of professional training, resulting in unprepared healthcare teams and facilities with inadequate infrastructure for larger bodies (^10^). Additionally, individuals living with obesity experience inequality in healthcare services, as professionals spend less time with them, provide less health education, are less likely to perform medical examinations and show less respect in their care compared to their treatment of individuals with lower body weight (^1^,^2^).

It is evident that individuals living with obesity experience negative emotions due to the pronounced weight stigma in healthcare settings. Patients feel devalued as a result of negative interactions with professionals, who often use derogatory language, exhibit dismissive nonverbal behaviour, fail to listen, and adopt a condescending attitude at various levels of healthcare (primary, secondary, and tertiary care) (^6^). And these settings frequently justify restricting access to services for individuals with obesity based on the mistaken belief that they are responsible for their weight and must demonstrate self-control by losing weight before accessing other treatments. Such justifications overlook the complexity of obesity, which involves biological, genetic, environmental, psychological, and social factors.

Public health also presents significant barriers concerning obesity, as governments fail to implement adequate actions to address the issue of weight stigma. Instead, many policies are stigmatizing, framing obesity as a matter of individual responsibility by emphasizing dietary changes and exercise while ignoring its multifactorial nature. While also putting obesity alongside other modifiable health risk factors such as smoking and alcohol consumption (^1^,^12^). Just as public health initiatives have historically placed blame on individuals for other chronic diseases, the same is now occurring with obesity, promoting the notion that people should be held accountable for their health (^1^,^2^). Furthermore, weight stigma is most pronounced among healthcare professionals who deprioritize government investment in obesityrelated actions and research, reinforcing the fact that stigma can influence decisions regarding obesity and the allocation of funding for studies in this crucial field (^4^).

Research has also reported that there is problematic portrayal and framing of obesity treatment where for instance, bariatric surgery is portrayed as an “easy way out” or that it is a “easy choice” and often public opinions including those of people living with obesity (^13^,^14^). This framing infers that weight management through behavioural modification, namely reduced energy intake and increased expenditure, is both harder and more worthy - and as such moralises obesity, compared to bariatric surgery. This inference ignores the complexity of obesity that means for many people living with obesity, behavioural modification would not lead to significant weight change. Moreover, the framing of bariatric surgery as easy is far from reality, given the very difficult preand post-surgery requirements including behavioural changes that for many will require life-long changes. Unfortunately, this framing continues to exist, with people living with obesity internalising this perception, which in some cases, may lead to shame and self-deprecation amongst those who choose this treatment option. With the new wave of medications for obesity receiving significant attention, there is emerging evidence that this harmful framing is being applied to these treatments. The potential benefits of these medications beyond weight loss including reduced risk of major cardiac events, is concerning. Akin to bariatric surgery, these medications also require significant long-term behavioural changes with evidence informing that when these medications are ceased, this often leads to weight regain (^15^,^16^).

## IMPACT OF WEIGHT STIGMA

It is well known that weight stigma is associated with psychosocial harm and is linked to an increased risk of depression, anxiety, and even suicide (^6^). Furthermore, weight stigma perpetuated by healthcare professionals may contribute to rising obesity rates due to the various levels of harm it can cause, leading to long-term mental and physical health deterioration, including worsening biochemical parameters associated with cardiometabolic risks and mortality (^9^). Prolonged exposure to the stress triggered by experiencing weight stigma can result in hormonal and physiological changes related to the onset of heart disease, stroke, and mental health diagnoses (^17^).

Experiencing weight stigma in healthcare settings can cause patients to experience stress induced by discrimination, leading to acute reactions that can negatively impact their health and the quality of their healthcare experience (^17^). These experiences are also associated with reduced engagement in healthcare and lower utilization of medical services, leading to avoidance and delays in seeking medical interventions (^9^). A significant factor to consider regarding stigma experiences in healthcare settings is the impact on internalized weight stigma, which refers to the acceptance of negative stereotypes and their application to oneself, resulting in self-devaluation due to weight. Internalized weight stigma is linked to feeling uncomfortable during physical examinations, perceiving medical judgment about one’s body, and increased avoidance of medical visits and health screenings (^5^).

Such avoidance and delays in seeking medical consultations occur due to fears of weight-loss discussions, over focus on weight as the cause of medical issues, concerns about weight gain, and anxiety over being weighed during appointments (^10^). These factors contribute to an increased risk of health deterioration, particularly in the prevention of chronic diseases that require long-term specialized treatment. For instance, women living with obesity exhibit high rates of avoiding medical appointments due to fears of body judgment, leading to a lack of preventive screenings for pelvic and breast examinations (^9^), as well as people present inadequate cancer screenings (^2^,^17^).

Many treatments are denied to individuals with obesity due to BMI cut-off points being used as acceptance criteria for procedures and treatments such as surgeries, transplants, fertility treatments, and gender-affirming care (^18^), driving inequity of healthcare. Moreover, weight stigma acts as a barrier in healthcare settings, impairing the prescription and availability of treatment alternatives for individuals with obesity, such as medications for obesity and metabolic surgery (^7^). Regarding bariatric and metabolic surgery, recommended for individuals who have been unable to lose weight through other treatments and experience significant health impairments due to obesity, there is often a delay in recommending surgery or, in some cases, it is not recommended at all. This is due to the belief that individuals must first demonstrate their commitment and engagement by losing weight on their own. This notion has been shown to not impact on weight loss following surgery resulting in American Society for Metabolic and Bariatric Surgery (ASMBS) not recommending presurgical weight loss due to insufficient scientific evidence of its benefits (^18^,^19^). A similar issue arises with the prescription of medications for obesity. Such perspectives are often reinforced by government actions aimed at reducing expenditures on procedures and medications, which ultimately result in the exclusion of individuals in genuine clinical need of appropriate treatment (^2^). Additionally, access to healthcare can be hindered due to weight-based discrimination in the workplace, which can result in denied job opportunities, reducing monthly income needed for health insurance and medical services. Regarding healthcare settings, patients can often struggle to find facilities that are structurally adequate and free from stigma, and are weight inclusive, where for instance, there are appropriately sized facilities and equipment including blood pressure cuffs, gowns and armless chairs.

## LANGUAGE MATTERS

Consistently reported in relation to stigmatising experiences in healthcare, is the language used by healthcare professionals and communication about weight. Research has reported that patients experience poor communication from healthcare professionals both when seeking care related and unrelated to obesity (^4^,^5^).

Several studies have examined preferences for weight-related terminology (^20^-^23^). highlighting that there are variations in opinions. Consistently, weight neutral terms are identified as the most preferred. Brown and Flint (^20^) also identified that there was a stronger dislike and a feeling of disgust for terminology that was accompanied by exacerbating verbs such as “super”, “extremely” and “morbidly”, and unlike other studies exploring weight-related preferences, their study also examined emotional response highlighting that sadness was associated with all terms. The least preferred terms were “super obese”, “chubby”, and “extra-large”, and for parents the least preferred for healthcare professionals to use about children with obesity were “fat”, “extra-large” and “extremely obese”. Studies examining terminology and language used by healthcare professionals highlight that neutral terms such as “weight” are the most preferred, that language used in consultations can impact the patient practitioner relationship and empathetic and compassionate communication can have a beneficial impact on patient engagement and future health seeking behaviour. The differences in terminology preferences suggest that where possible, healthcare professionals elicit the patients’ preferred weight-related terminology.

Healthcare professional communication about obesity has been reported to be unhelpful, and in some instances stigmatising and derogatory. This includes communication within patient-practitioner consultations where rhetoric is intentionally stigmatising reflecting stereotypes relating to lacking willpower and gluttony (^24^), whilst others occur unintentionally as a result of a lack of communication training.

## ADDRESSING WEIGHT STIGMA IN HEALTHCARE

In response to the accumulating evidence of the pervasiveness and impact of weight stigma, there have been calls for interventions, with many intended to reduce weight stigma amongst healthcare professionals. Various types of interventions have been trialled including education, evoking empathy, conditioning, weight inclusive approaches and group dynamic based tasks to foster collaboration. Many of these interventions have reported limited success, and where reductions have been reported, these effects have often dissipated back to baseline overtime. The Health at Every Size (HAES) model represents a weight-inclusive approach that shifts focus away from weight and weight loss as primary health metrics, instead advocating for a comprehensive, multidimensional understanding of wellbeing. Research evidence indicates this paradigm demonstrates effectiveness in improving cardiometabolic outcomes, enhancing quality of life indicators, and increasing treatment compliance rates (^25^,^26^). Education about the aetiology and complexity of obesity that informs of the evidenced wide-ranging factors that may lead to obesity, including the factors either partially or solely outside of a person’s control (e.g., genetic, environmental) as well as education about weight stigma, appear to have the greatest effect, albeit limited (^27^). For instance, Velazquez and cols. (^28^) reported that a 4-hour education-based intervention was effective at reducing weight bias amongst healthcare professionals with the effect sustained at 4- and 12-months post-intervention.

Educational and training for healthcare professionals have emerged, with a focus on improving understanding of the aitiology and complexity of obesity, weight management interventions including behavioural, pharmacotherapy and surgery based on a person’s and weight and health status, and importantly, training specifically to address weight stigma, communication and behaviour change techniques (^29^-^31^). Guidelines for healthcare professionals have also been created by professional organisation and societies for members (^32^,^33^), and by researchers (^1^,^34^). Research highlights the potential impact of stigmatising language in consultations that for instance, lead to negative emotional response that does not support behavioural changes (^20^). Stigmatising language can impact the patientpractitioner relationship and may lead to healthcare avoidance, opportunities to enhance communication techniques and where appropriate to elicit preferred language from patients in consultations is advised.

## SUMMARY AND STRATEGIES TO REDUCE WEIGHT STIGMA IN HEALTHCARE

Addressing weight stigma is not going to be quick or easy given how ingrained it is in society. However, changes are needed, and we must embrace these changes given the substantial evidence about the detrimental effects of weight stigma experiences, with even small changes like the ones listed below having the potential to substantially improve the experience of people living with obesity within healthcare. These strategies address the limitations of previous efforts where for instance, education about obesity that has shown promise in reducing weight stigma attitudes and reduced attribution of blame also involves practical solutions to implement change. First, it is imperative that people living obesity are included and play a key role in the design and development of obesity related policy, services and research, which is currently rarely happening. Progress has been made, particularly in research, where research authorities and councils have stipulated patients and public involvement. Second, given the pervasiveness of weight stigma that means most people are exposed to weight stigma on almost a daily basis, we must be prepared to address our own biases and potentially behaviours; weight stigma may be implicit and intentional. Third, within healthcare, there is a need for improved empathy and compassion towards people living with obesity who have and, in some instances, continue to face inequity in access to care and discrimination in healthcare settings (^1^). Improvements in empathy are needed both in the consultation room through patient-practitioner interactions and in decisions about care. Fourth, appreciate the impact of weight stigma experiences and that addressing weight bias internalisation may be an important first step as part of any form of care package. Fifth, create weight inclusive healthcare environments that appropriate for people of all body sizes. This may include equipment and facilities such as armless chairs and appropriately sized gowns. Sixth, the adoption of a weight-inclusive narrative (as opposed to a weight-normative approach) in healthcare, research, and individual care should be prioritized. Seventh, increased training, education, and updated guidelines for healthcare professionals are essential to ensure alignment with current evidence on obesity. This includes highlighting its multifactorial origins, particularly the influence of environmental determinants, and moving beyond individual-centric blame narratives. Finally, strengthening psychosocial support systems and economic accessibility is crucial, as these factors significantly impact an individual’s ability to obtain comprehensive healthcare, engage with supportive environments, and achieve overall wellbeing. Consequently, governments must implement coordinated public policies to guarantee equitable healthcare access and the necessary conditions for a healthy life.

## SUMMARY

In this narrative review, we provide an overview of the key issues relating to weight stigma and its impact on people living with obesity. Weight stigma in healthcare is rife with evidence demonstrating that this is a key setting where people living with obesity experience it. Stigmatising communication from healthcare professionals has and continues to be reported, where blame and individual responsibility frequently cited despite the evidenced complexity of obesity that goes beyond individual agency. As such, weight stigma interventions are needed that address the myths and misconceptions of obesity, that are long-term and aligned across the system given that weight stigma is evidenced across society, and that reinforce compassion and empathy.

## Data Availability

datasets related to this article will be available upon request to the corresponding author.
